# Dental Diagnosis and Treatment Assessments: Between X-rays Radiography and Optical Coherence Tomography

**DOI:** 10.3390/ma13214825

**Published:** 2020-10-28

**Authors:** Ralph-Alexandru Erdelyi, Virgil-Florin Duma, Cosmin Sinescu, George Mihai Dobre, Adrian Bradu, Adrian Podoleanu

**Affiliations:** 1 Doctoral School, Polytechnic University of Timisoara, 1 Mihai Viteazu Ave., 300222 Timisoara, Romania; ralph.erdelyi@student.upt.ro; 2 3OM Optomechatronics Group, “Aurel Vlaicu” University of Arad, 77 Revolutiei Ave., 310130 Arad, Romania; 3 Research Center in Dental Medicine Using Conventional and Alternative Technologies, School of Dental Medicine, “Victor Babes” University of Medicine and Pharmacy of Timisoara, 9 Revolutiei 1989 Ave., 300070 Timisoara, Romania; minosinescu@gmail.com; 4 Applied Optics Group, School of Physics, University of Kent, Canterbury CT2 7NR, UK; gd@kent.ac.uk (G.M.D.); a.bradu@kent.ac.uk (A.B.); a.g.h.podoleanu@kent.ac.uk (A.P.)

**Keywords:** biomedical imaging, dental medicine, X-ray radiography, Optical Coherence Tomography (OCT), Cone Beam Computed Tomography (CBCT), dental cavities, biocompatible materials, optical measurements, quantitative assessments

## Abstract

A correct diagnosis in dental medicine is typically provided only after clinical and radiological evaluations. They are also required for treatment assessments. The aim of this study is to establish the boundaries from which a modern, although established, imaging technique, Optical Coherence Tomography (OCT), is more suitable than the common X-ray radiography to assess dental issues and treatments. The most common methods for daily-basis clinical imaging are utilized in this study for extracted teeth (but also for other dental samples and materials), i.e., panoramic, intraoral radiography, and three-dimensional (3D) cone beam computed tomography (CBCT). The advantages of using OCT as an imaging method in dentistry are discussed, with a focus on its superior image resolution. Drawbacks related to its limited penetration depth and Field-of-View (FOV) are pointed out. High-quality radiological investigations are performed, measurements are done, and data collected. The same teeth and samples are also imaged (mostly) with an in-house developed Swept Source (SS)-OCT system, Master-Slave enhanced. Some of the OCT investigations employed two other in-house developed OCT systems, Spectral Domain (SD) and Time Domain (TD). Dedicated toolbars from Romexis software (Planmeca, Helsinki, Finland) are used to perform measurements using both radiography and OCT. Clinical conclusions are drawn from the investigations. Upsides and downsides of the two medical imaging techniques are concluded for each type of considered diagnosis. For treatment assessments, it is concluded that OCT is more appropriate than radiography in all applications, except bone-related investigations and periodontitis that demand data from higher-penetration depths than possible with the current level of OCT technology.

## 1. Introduction

Dentistry has been evolving fast in the last few decades through technological advances in both diagnosis and treatment [1,2,3]. For diagnosis there are several types of medical imaging techniques available, including X-ray radiography, laser-based pens for the detection of cavities, as well as Optical Coherence Tomography (OCT) [4,5,6,7].

The most common methods for daily-basis clinical imaging in dental medicine are intraoral and panoramic radiography, as well as three-dimensional (3D) Cone Beam Computed Tomography (CBCT). One of their drawbacks is the patients’ concern with being exposed to X-ray radiation, which is ionizing and harmful for living tissue. In this respect, the radiation dose must be properly calculated by technical personnel for every method [8], while X-ray units and investigations must be improved to reduce the radiation dose [9]. This is ideally achieved without losing imaging performances, as accurate high-quality images with high resolution, good contrast, and no artefacts are mandatory to correctly diagnose a patient or to assess a performed treatment. Currently, digital X-ray units (equipped with appropriate sensors and dedicated software) can be optimized to enhance, process, and analyze in-depth obtained images. X-rays techniques are limited in resolution: around 127 μm for panoramic, 144 μm for intraoral radiographies, and 75 μm for CBCT [9]. Some dental issues cannot thus be correctly assessed, whatever the type of radiography nor using visual observation. In consequence, other medical imaging techniques are necessary for clinicians to allow them to provide a quality treatment.

OCT is such an imaging technique that can be utilized to diagnose dental affections [10,11,12,13,14,15,16,17] and to assess performed treatments [14]. OCT is not yet a common imaging method in dentistry, although it has imposed itself in ophthalmoscopy [4,18], but also for skin investigations [19] (in conjunction with confocal microscopy) and endoscopy [20,21]. As OCT uses low-power IR laser radiation, it is entirely non-invasive, with the advantage of avoiding patients’ exposure to radiation—in contrast to X-ray techniques. However, image resolution in OCT is much better, with common values of 8 μm for the axial resolution reported in the current manuscript and values of 2 μm (both axial and lateral) as state-of-the-art [22], while sub-micrometer values have also been explored (but in VIS, not in IR, therefore not applicable in dentistry, as the penetration depth would be too small) [23]. While the possible utility of OCT for different Dental Medicine investigations has been demonstrated by numerous studies, to our knowledge a study on establishing clear suitability of this technique versus X-rays considering the existing range of dental issues and applications is still necessary.

The aim of this work is to contribute in this direction, to establish which conditions affecting hard tissue in the oral cavity can be investigated only with X-rays, with both X-rays and OCT, as well as only with OCT. The advantages and drawbacks of each technique must also be considered. Comparing OCT with (common) radiography, its clear drawback is the much lower penetration depth. This is inversely proportional to the density of the material being investigated, therefore limited to at most 1.5 mm for hard dental tissue or bone. On the other hand, OCT images reveal dental issues at an earlier stage than radiographs due to their superior resolution [17].

Quantitative assessment is another important rationale for such a comparative study. Measurements with dedicated software can be used to serve investigation of cavities, secondary cavities, length of the root canal, or periodontitis. Without quantitative tools, images delivered by any technique can only serve qualitative analysis. Thus, the image of a cavity, for example (as delivered by OCT), must be processed and analyzed to provide a quantitative information [24]. To compare X-rays techniques and OCT from the point of view of the precision of such assessments is another aim of this work. To fulfill its scope, a range of in vivo investigated clinical cases in the oral cavity and ex vivo assessments (the latter on extracted teeth) are considered in the study.

Finally, to compare the capabilities of the two techniques, investigations after a dental treatment are made, for both cavities and dental crowns.

## 2. Materials and Methods

### 2.1. Radiography

For this study, several extracted teeth were gathered from the *Dental Experts Clinic*, Timisoara, Romania, following the Ethical protocol of the Clinic, with the written consent of the patients. All teeth were extracted during different treatments, and not for the sole purpose of this study. While such samples are ex vivo X-ray imaged (see Section 3.2 and Section 3.3), other such investigations are performed in vivo in the above clinic, during clinical investigations, on bone and teeth in the oral cavity (see Section 3.1).

**Figure 1:**
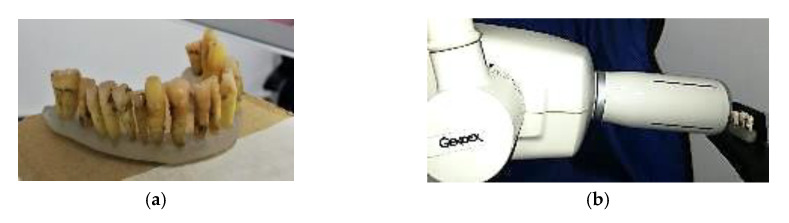
(**a**) Prepared teeth for X-ray investigations; (**b**) teeth positioned in the X-ray unit Gendex Oralix (Danaher Corporation, Washington DC, USA), ready for exposure.

The radiological investigations with additional measurements are performed in the clinic using two radiological units: Planmeca ProMax 3D Plus (Planmeca, Helsinki, Finland) for panoramic radiography, and 3D CBCT, Gendex Oralix (Danaher Corporation, Washington, DC, USA) for intraoral radiography [25]—Figure 1.

The maximum resolution achieved with both X-ray units was 75 µm, after the optimizations described in detail in [9]. The protocol for obtaining such high-quality radiographs was optimized to comply with the *As Low As Reasonably Achievable* (ALARA) protocol [26]. This means that the X-ray unit provides the highest possible quality radiography, exposing the patient to the smallest possible amount of radiation. Intraoral radiography is performed at 68 kV and 9 mA for an exposure time between 0.5 and 1 s. Panoramic radiography and 3D CBCT have unchangeable exposure time of 15 s and 5 s, respectively. X-ray tube settings for panoramic radiography are 72–73 kV and 11 mA, while for 3D CBCT they are 90 kV and 14 mA, with an additional ultra-low dose (ULD) protocol.

To obtain high-quality images and to improve the radiographs, or to assess issues of treatments using them [27,28], each X-ray unit is equipped with additional computing power. The Planmeca X-ray unit is part of a system with two additional PCs, all linked in a private network: the first one works as a server and for image reconstruction, and the second one for image processing. The image reconstruction PC has an Intel Core i5 (6th generation) CPU, 16 GB RAM, an x64 based operating system, two memory disks (1 SSD with 128 GB storage space and 1 HDD with 1 TB storage space), a dedicated GPU with minimum 2 GB RAM, and a LAN connection. This PC collects the information from the sensor [9], processes and converts it into a raw 2D or 3D image. The image processing PC has an Intel Core i7 (6th generation), 16 GB RAM, an ×64 based operating system, three memory disks (1 SSD with 256 GB storage space and 2 HDD with 1 TB storage space each, connected in a RAID1 configuration), a dedicated GPU with minimum 2 GB RAM, and it should have 2 LAN connections. RAID1 means that the same information is written on both HDDs and it is protected if an HDD is damaged. Thus, all data are safe and remain stored on the other HDD.

### 2.2. Optical Coherence Tomography (OCT)

**Figure 2:**
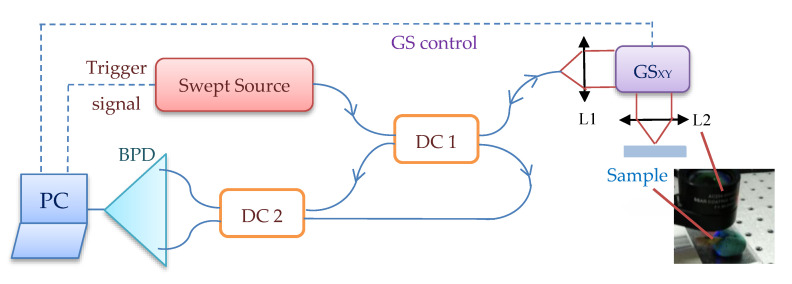
In-house developed MS/SS-OCT system using a single interferometer at two stages. Components: Swept Source; DC_1,2_, single mode directional couplers (20/80 and 50/50, respectively); GS_XY_, dual axis galvanometer scanner; L1,2, achromatic lenses; BPD, balanced photo-detector; PC, personal computer.

OCT investigations have been performed mainly using an in-house developed Swept Source (SS) OCT system, Master-Slave enhanced [7], (schematic diagram shown in Figure 2), at the “Aurel Vlaicu” University of Arad, Romania. It includes a 50 kHz laser source swept in frequency (Axsun Technologies Ltd., Billerica, MA, USA), with a centre wavelength of 1310 nm and a sweeping range from 1256.6 nm to 1362.8 nm. Its output optical beam (optical power 18 mW at the output of the laser) is directed towards an 80/20 directional coupler which conveys 20% of the source optical power towards the sample via a dual axis 2D galvanometer scanner (GS) [29]. The back-scattered light from the sample is guided back along the same path and is subsequently combined at coupler DC2 with the reference light. Each of the two DC2 arms leading to the balanced photodetector BPD (Santec Model BPD-200 DC, (Komaki-City, Japan)) carries interference light resulting from the recombination of sample and reference light. They are converted into two electronic signals in opposite phase. The signal resulting from the difference operation is stripped of its DC slow varying component and its ac pulsates at an amplitude twice that of each photodetector signal due to the interference between sample and reference beams. This signal is further digitised by a 12 bit, 500 MS/s waveform digitizer model ATS9350 (Alazartech, Quebec, QC, Canada), converted to greyscale, put in a form suitable for viewing, and displayed using an in-house developed software, implemented in LabVIEW 2013, 64 bit. The same program also drives the 2D GSs via a data acquisition board model PCI 6110 (National Instruments, Austin, TX, USA). The acquired channeled spectra are used to build a 3D OCT image and produce C-scans/*en-face* images (situated at a certain depth in the sample), using the Master-Slave (MS) protocol [7]. This protocol allows for obtaining *en-face* images directly, without performing volumetric reconstructions first, using B-scans, as in conventional SS-OCT. The axial resolution provided by the instrument is 10 µm measured in air.

The OCT system use optical power at conservative level, as employed in imaging the retina, a few mW maximum, although larger power could be tolerated. At the level of safety values for the retina, sensitivity is 85–92 dB at 100 kHz line rates. For the Axsun source used in the setup in Figure 2 (with 1310 nm and 50 kHz), a sensitivity >97 dB is typically obtained with 3.6 mW optical power on the sample. There are numerous reports showing that MHz line rates are feasible within the power limitation due to safety, a few mW, hence similar speeds should be achievable in the applications concerned to this report, with immediate calculation in degrading the sensitivity proportional with the speed increase.

While in [7] the principle of MS has been first introduced using two interferometers, Master and Slave, the same study illustrated the implementation of MS using a single interferometer at two stages. As in practice, instead of a second interferometer (the Master one) a storage of channeled spectra can be employed, this is the main way MS is performed in this study as well.

For some of the results presented in Section 3.4 on teeth and dental crowns, two other in-house developed OCT systems were used. These were a Spectral Domain (SD) and a Time Domain (TD) one, described in [30,31], respectively. Samples were extracted from patients attending the “Victor Babes” University of Medicine and Pharmacy of Timisoara, Romania, following an approved Ethical protocol and after their written consent.

### 2.3. Characterization of Samples

Several methods have been employed to characterize the samples of each group from different points of view, as briefly presented in the following protocol.

Extracted teeth are first analyzed with X-ray techniques since the radiology equipment is also located in the dental clinic that has provided (most of) the teeth for this study. After the teeth are extracted, they are cleaned and prepared for investigations. All types of radiographies are performed with all the available equipment in the clinic (common for such a medical environment): intraoral radiography, panoramic radiography, and 3D CBCT. In Figure 1, examples of teeth prepared for investigations are shown. Romexis Viewer (Planmeca, Helsinki, Finland) is the software utilized to assess cavities or other dental issues. This is equipped with a toolbar that allows precise measurements of dental aspects, even for images imported from other sources [32]. This is a novel approach of this study, as most OCT studies are usually performed using an open source image processing software, ImageJ (Wayne Rasband, NIH/LOCI, University of Wisconsin). In this study, to make sure that differences in quantitative assessments are only related to the performance of the techniques and not to software characteristics, the same software, Romexis Viewer is utilized.

After the image is provided by the X-ray unit or imported from another source (i.e., the SS-OCT system), a calibration step is mandatory. This implies a correlation between the number of pixels and the area of the surface, performed with the measurement toolbar, which also serves for calibration, as well as for angles and lengths measurement [32]. However, we must note that, even if a software is a trustworthy tool for accomplishing a correct assessment of an issue, it cannot surpass limitations of the imported image’s resolution. Thus, the software cannot be used to analyze details that cannot be observed on radiographs.

OCT investigations of different samples have been done using the MS/SS-OCT system in Figure 2, as well as (for a few samples) the SD and TD systems pointed out in the previous subsection. Teeth need no preparation for OCT investigations, as they do not need any for X-ray radiography as well. A total of 500 OCT B-scans have been obtained for each sample from different lateral locations. They have been further processed and analyzed with ImageJ, being rendered into a 3D image/volumetric reconstruction. Where comparisons between radiography and OCT have been necessary, both B-scans and 3D OCT images have been then imported in Romexis Viewer, for measurements and a parallel study with radiographs.

## 3. Results and Discussion

### 3.1. Radiography-Oriented Dental Investigations

**Figure 3:**
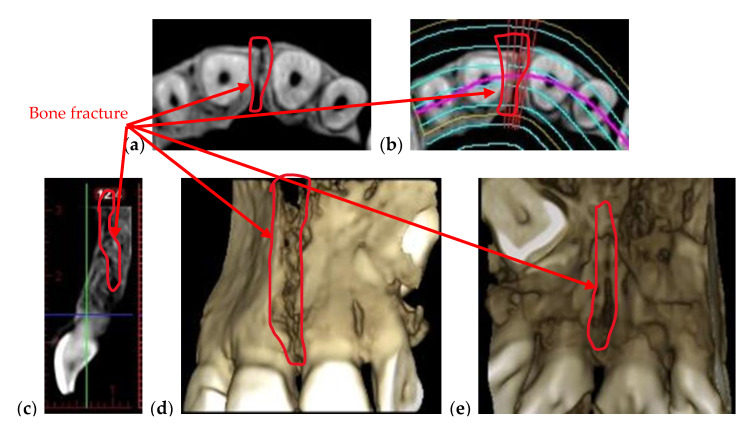
3D CBCT of a fractured maxillary in different views: (**a**) axial view; (**b**) axial view indicating the position of the fracture; (**c**) sagittal view; 3D reconstruction, (**d**) frontal and (**e**) posterior view, previously detailed in [28]. Patient V.L., female, age 29 years, diagnosed with a crack in her maxillary bone caused by a head trauma.

Radiography is the daily-basis medical imaging technique in dentistry, therefore it is difficult to select dental disorders that are visible only using this technique. From its variants, panoramic radiography is the first method that can be (and is usually) performed, as it has the advantage of providing an ample perspective of the full mouth of a patient in (only) a few seconds of investigation,

For a correct diagnosis, patients must be checked both clinically and radiologically. For bone diseases (implying periodontitis or fractures) or for bone assessment, panoramic radiographs are not necessary for density or post-operator investigations. A 3D CBCT has to be performed in such situations because in addition to a qualitative assessment, it offers volumetric information. An example of a CBCT investigation performed in the clinic for a fractured maxillary is presented in Figure 3.

One can remark that, in such cases, OCT cannot be of use, as the necessary depth of the investigation is beyond its capability. However, the crack in Figure 3 is also large enough for the CBCT resolution to be sufficient to spot and assess its dimensions.

**Figure 4:**
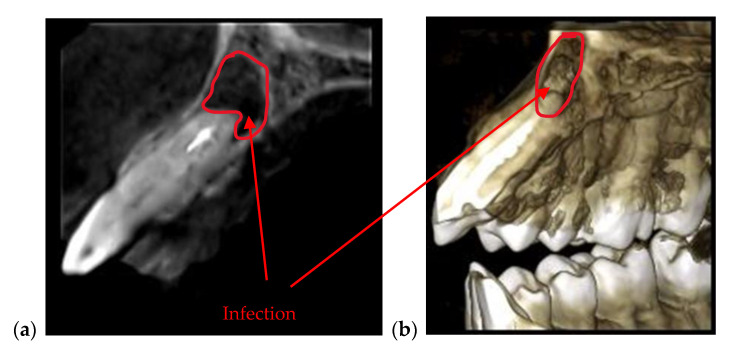
(**a**) Sagittal view and (**b**) 3D CBCT reconstruction of an infection formed at the tip of a tooth. Patient C.B.G., female, age 37 years, diagnosed with dental abscess.

3D CBCT is also recommended when it is important to assess the tip of the tooth’s root, as presented in Figure 4. In such cases intraoral and panoramic radiographs do not offer reliable information because they provide 2D images, and if a dental infection is behind the tooth, it is not visible. Such investigations are beyond the penetration capability of OCT as well.

**Figure 5:**
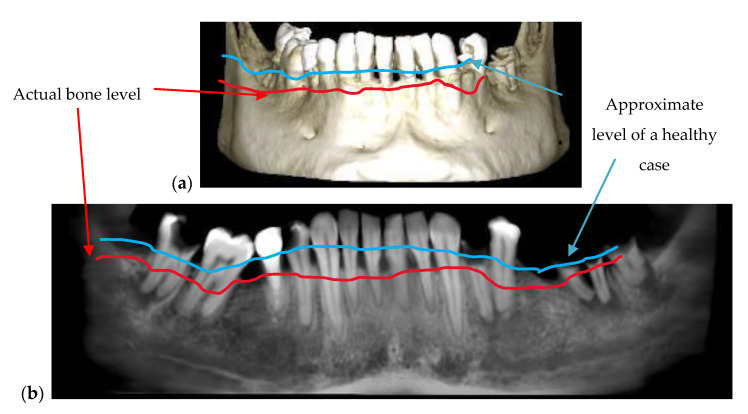
Periodontitis disease observed on a 3D CBCT reconstruction (**a**) and on a panoramic radiography (**b**). Patient C.O., male, age 34 years, diagnosed with periodontitis, alongside other dental issues such as cavities and dental abscesses.

The periodontitis disease, in its advanced stages, can be diagnosed using any type of radiography. Figure 5 is an example of periodontitis disease observed on both 2D and 3D radiography. The red line represents the actual level of bone that is affected by the disease and the blue line represents approximately the level where the healthy bone should be. The issue is to detect it in (very) early stages, if possible, to apply appropriate treatments before the gingiva has begun to retreat.

While a range of methods can be used for diagnosis, from periodontal probes [33] to 3D CBCT with high resolution and low radiation dose [34,35], different biomaterials such as nanoparticles are considered to improve the performance in detecting and measuring periodontal pockets [36]. In this respect, making successive OCT investigations of the same area every 6 months can be relevant for the clinician regarding the success of the treatment. This can be in a similar way to using (ex vivo) holography on models to make such assessments [37]. A comparison of these two techniques in this respect is the subject of future work.

**Figure 6:**
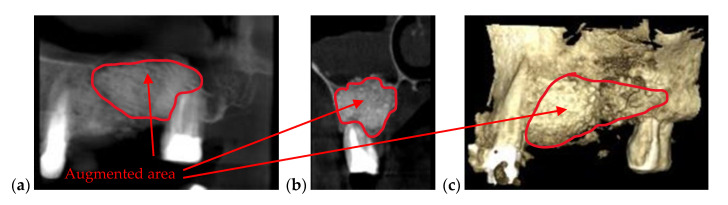
(**a**) Panoramic view, (**b**) sagittal view, and (**c**) 3D reconstruction of an augmented bone obtained after a segmental 3D CBCT with a FOV of 5 × 5 cm. Patient P.P., male, age 46 years, diagnosed with severe periodontitis.

Augmented bone is necessary to provide enough jawbone volume for a successful dental implementation [38,39]. As various diseases (including trauma, cancers, or osteoporosis) occur, the alveolar ridge must be augmented (Figure 6), because there is not enough bone left to use implants. Allografts or autografts may be utilized, although the former may transmit certain diseases, while the latter involve additional clinical procedures and increase morbidity. Alternate materials such as bioceramics are developed for such scaffolds [40], while procedures such as photo-biomodulation/Low-Level Laser Therapy (LLLT) are demonstrated to accelerate new bone formation when additional bone particles are utilized to stimulate bone regeneration. For the latter, we have used OCT to demonstrate the positive impact of LLLT on new bone formation [41]. The advantage of OCT is its capability to monitor the process in vivo, non-invasively (in contrast to micro-CT or the gold standard of histopathology [42]), and with higher resolution than radiography. The OCT’s drawback in this case as well is its lower penetration depth and Field-of-View (FOV), (the latter imposing mosaicking images [43] or segmenting investigations [41]), while radiography has both enough penetration depth and FOV to assess the results of the bone-augmentation process, as shown in the example considered in Figure 6. Because of this disease, the patient lost several teeth that cannot be replaced with dental implants because of the patient’s insufficiency in bone quantity and density. To make possible the surgery of implants insertion, the patient was subjected to an additional surgery of bone augmentation. The augmentation was made with Geistlich Bio-Oss (Wolhusen, Switzerland), which is a natural bone mineral of bovine origin that is available as granules of spongious bones in an applicator.

### 3.2. OCT-Oriented Dental Investigations, Compared with Radiographs

**Figure 7:**
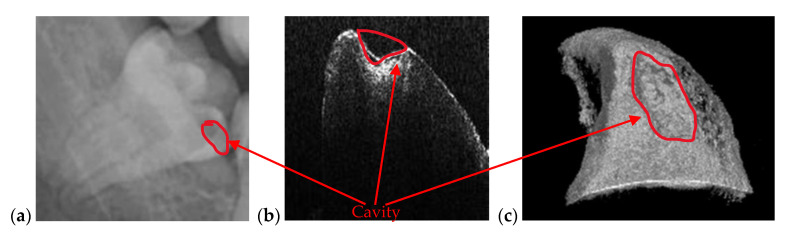
(**a**) Cavity of a third molar from the fourth quadrant assessed on a section cropped from a panoramic radiography. OCT investigation on the tooth extracted for medical purposes after performing the radiography: (**b**) B-scan and (**c**) 3D reconstruction. Patient E.M., male, age 23 years, diagnosed with a cavity on the smooth surface (lateral side) of the third molar, with the following remarks on the clinic condition: the cavity appeared because the third molar is not in a correct position and in that area, between the second and the third molar, the patient cannot perform a full cleaning of the tooth.

**Figure 8:**
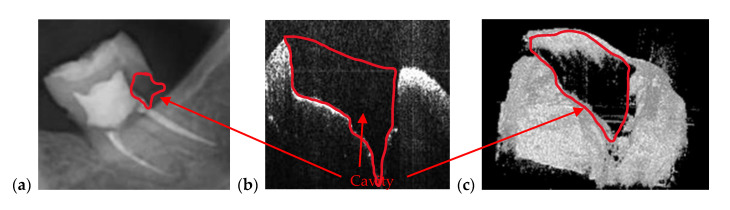
Cavity of a second molar from the third quadrant assessed on (**a**) a section cropped from a panoramic radiography. OCT investigation on the tooth extracted for medical purposes after performing the radiography: (**b**) B-scan and (**c**) 3D reconstruction. Patient M.N., female, age 29 years, diagnosed with a cavity on the smooth surface of the tooth and one of the tooth’s root. The latter is so large because there are two cavities connected with each other: The first one is a recurrent cavity that appeared under the filling because of an endodontic treatment; the second one appeared because of the receding gingiva and mandibular bone, which has left the tooth exposed.

**Figure 9:**
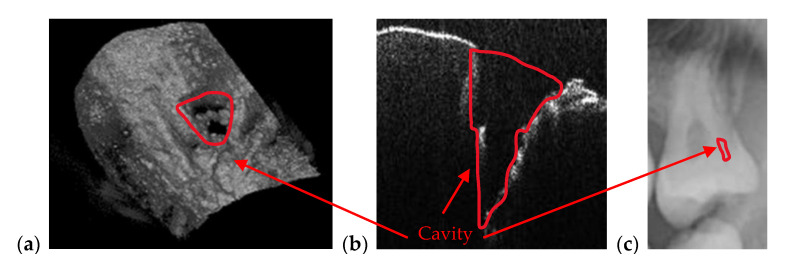
Cavity of a third molar from the second quadrant assessed on: (**a**) an OCT 3D reconstruction and (**b**) an OCT B-scan, both performed on the tooth extracted for medical purposes after performing the radiography; (**c**) a section cropped from a panoramic radiograph. Patient C.M., male, age 24 years, diagnosed with a small cavity, with the following remarks on his clinic condition: the tooth did not pass completely from the gingiva, but food, as well as degenerative factors entered between tooth and gingiva. This is the reason the cavity has appeared.

**Figure 10:**
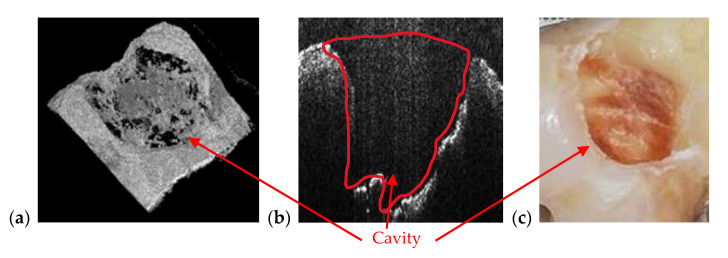
Cavity of a third molar from the second quadrant assessed on (**a**) an OCT 3D reconstruction and on (**b**) an OCT B-scan. Image (**c**) is a photograph of the cavity. Patient R.E., male, age 27 years, diagnosed with a large cavity formed at the border between a dental crown and the tooth’s root. The reason for this cavity is the impossibility of the patient to clean that area because of the gingiva and the inner cheek thickness.

**Figure 11:**
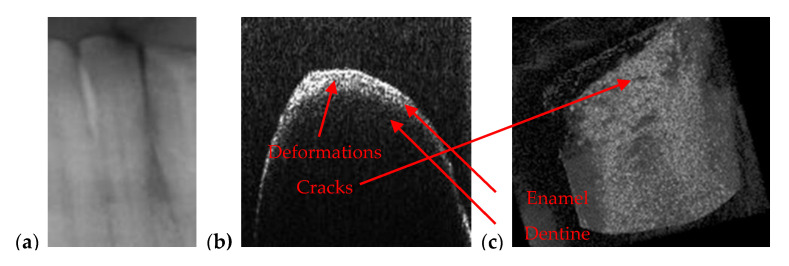
Enamel deformations of an incisive tooth from mandible that cannot be observed on a section cropped from a panoramic radiography (**a**) but can be clearly remarked both on (**b**) OCT B-scans and on (**c**) a 3D OCT reconstruction (performed on the tooth extracted for medical purposes after performing the radiography).

**Figure 12:**
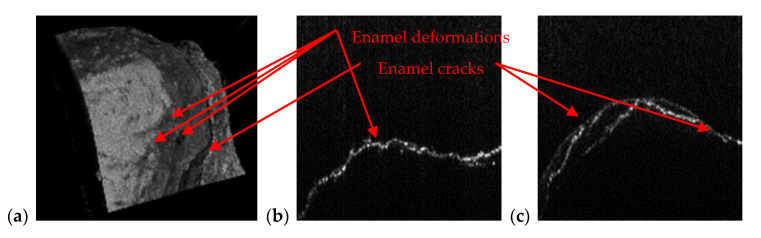
Enamel deformations and cracks of an incisive tooth observed on (**a**) OCT 3D reconstruction, as well as on (**b**,**c**) different B-scans.

**Figure 13:**
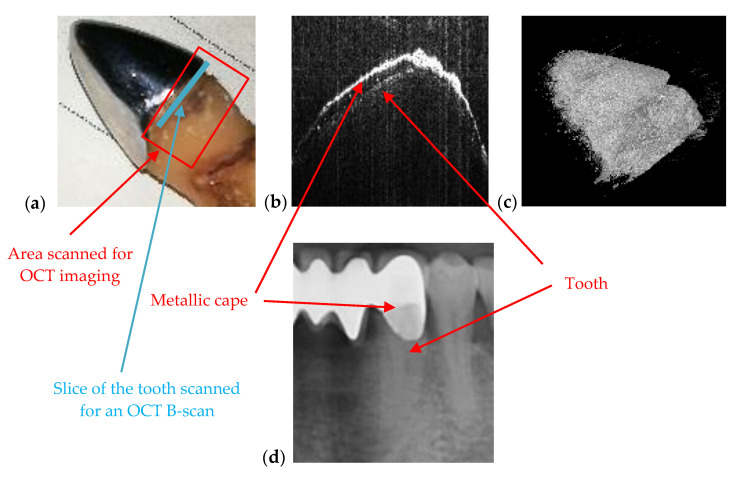
OCT used to check the adaptation of metallic cape on the tooth: (**a**) photo of the tooth with cape, (**b**) OCT B-scan showing both the tooth and the metallic cape, (**c**) 3D reconstruction, and (**d**) section cropped from a panoramic radiography. Patient S.S., female, age 57 years, diagnosed with multiple abscessed teeth and periodontitis.

**Figure 14:**
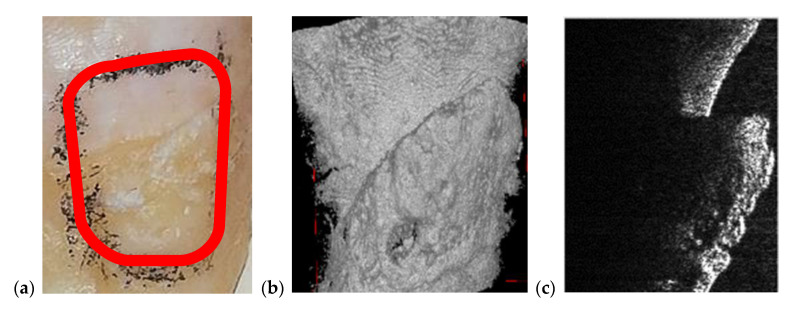
Deep crack in the enamel layer observed on (**a**) part of the photo of an extracted tooth, as well as on (**b**) the 3D OCT reconstruction of the selected area. (**c**) OCT B-scan showing the shape of the crack. If necessary, the dimensions (especially the depth) of the crack can be accurately measured on such a B-scan. Patient C.T., male, age 23 years, diagnosed with several dental problems on a third molar (i.e., gingiva inflammation, abscess, cavities, and wrong growth of the tooth).

Cavities assessment, enamel or dentine issues such as cracks or demineralization, adaptation of dental fillings or crowns are examples of several dental affections that can be better assessed on OCT images than on any type of radiography, as documented by different groups [10,11,12,13,14,15,44,45,46], including ours [17,28,47]. The investigations in Figure 7, Figure 8, Figure 9, Figure 10, Figure 11, Figure 12, Figure 13 and Figure 14 on examples of such dental issues prove that OCT images allow for a more accurate diagnosis than radiographs in several situations, where resolution is paramount, and the penetration depth of OCT is enough.

Figure 7 is an example of the superior resolution and contrast of OCT images. This can be best seen on a volumetric/3D reconstruction in Figure 7c, but also on a well-chosen cross-section/B-scan in Figure 7b. In contrast, in the radiograph in Figure 7a, the (large) dental cavity can barely be spotted. As demonstrated in the following section, such cavities can be exactly measured on OCT images, while on radiographs they can only be observed. Similar remarks can be made regarding the examples presented in Figure 8 and Figure 9.

In the example in Figure 10, the precision of OCT technique when it comes to imaging dental cavities can be remarked by analyzing Figure 10a,b. The margins of OCT 3D rendering in Figure 10a is 1:1 with the margins obtained from the photography in Figure 10c. This is one of the reasons that makes OCT the appropriate medical imaging technique when it comes to investigate cavities. Alongside its superior accuracy, OCT is radiation-free. Regarding the acquisition speeds, they are from 1 to 15 s for different types of radiographs (as pointed out in Section 2.1), while for OCT they are much faster, usually in milliseconds-for a common individual scan, with the FOV corresponding to an area of up to 3 × 3 mm^2^. If mosaic OCT images are performed [43], the acquisition time can be longer, but less than 1 s in all situations. For the MS enhanced SS-OCT system used in this study (Figure 2), OCT imaging is also performed in real time, with no post-processing of images.

Beside cavities, OCT is capable to detect abnormalities at the level of enamel and dentine. As demonstrated in the examples in Figure 11, Figure 12, Figure 13 and Figure 14, because of its high resolution, OCT images reveal dental issues such as enamel deformations or cracks. Figure 11b,c reveal enamel deformations at the cusp of the tooth and some small cracks on the smooth surface of the tooth. Figure 11a is a section of a panoramic radiography, and the issues visible on OCT images are not spotted at all on the panoramic radiography.

Figure 12 is also revealing enamel deformations and cracks on a smooth surface of an incisive tooth. In this case, these dental aspects are more visible using OCT than in the case in Figure 11; in the panoramic radiography they are not even spotted. As a remark, compared to such classical structure-oriented OCT, polarization sensitive (PS) OCT can provide much higher contrast on enamel deformations or demineralization in the enamel [48]. Therefore, applying PS OCT for such dental issues can be a valuable direction of future work. Images of the teeth from Figure 11 and Figure 12 belong to the same patient, T.C., male, age 34 years, diagnosed with advanced periodontitis.

Another situation where OCT is better suited than X-ray imaging is when the adaptation of dental crown on the abutment prepared tooth must be checked. Figure 13 is showing an example of a tooth with a metallic cape. The adaptation of this cape is visible only on OCT images (Figure 13b). Figure 13d is a section cropped from a panoramic radiograph, and one can see that on this image the adaptation cannot be assessed. In Figure 13b, one can observe the metallic layer and the tooth because the OCT B-scan is obtained at the junction between these two components, marked with the blue line in Figure 13a. In the area where the metallic cape is scanned, one cannot see the tooth because the IR laser radiation specific to OCT does not pass through metallic surfaces. Figure 13c is the OCT 3D reconstruction of this selected area marked in Figure 13a.

In Figure 14a, a deep crack in a tooth is imaged. While the crack can be observed visually, in Figure 14a its depth can be assessed quantitatively using OCT images, choosing the appropriate B-scan, Figure 14c, from the 3D OCT reconstruction of the zone of interest, Figure 14b.

### 3.3. Measuring Dental Cavities on Both OCT Images and Radiographs

The aim of this section is to utilize OCT images to see if one can diagnose clear margins of cavities. These results are compared with those obtained with radiographs. One must point out in this respect that, even if the resolution is the same for any type of radiography, there are differences in the details that can be observed on the acquired images. For example, a small cavity cannot be diagnosed exactly on a panoramic radiography, but an intraoral radiography delivers more detailed information. The advantage of the intraoral radiography over panoramic radiography in the case of a small cavity is related to the fact that for the former the focus is on that part of the mouth where the tooth with a specific affection is located [27].

**Figure 15:**
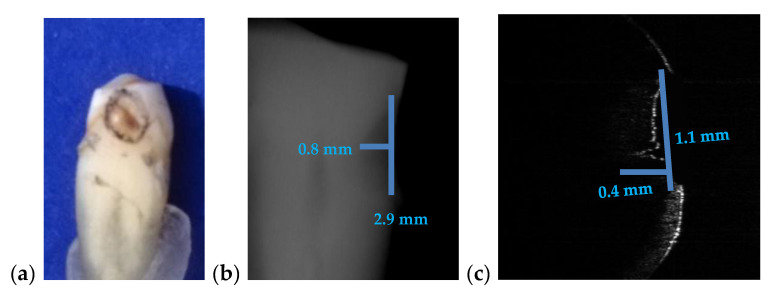

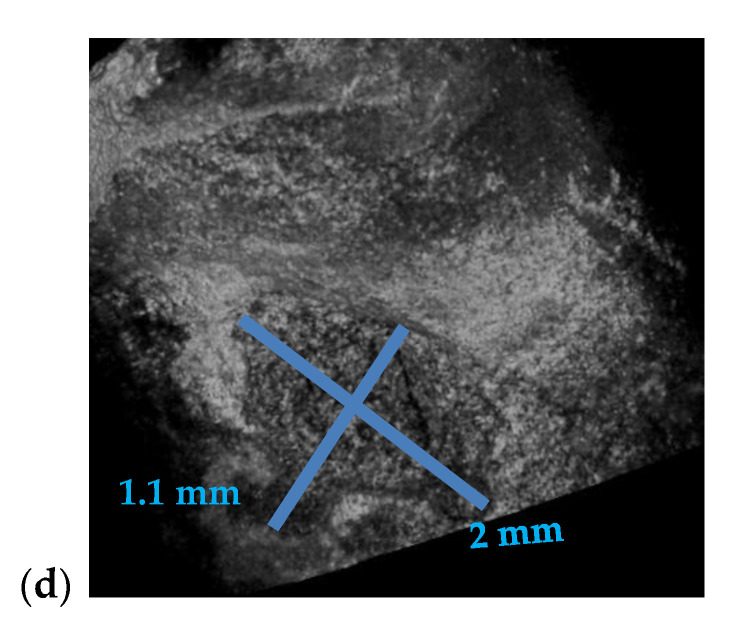
(**a**) A premolar tooth, with an area marked for OCT investigations; (**b**) section cropped from an intraoral radiograph with a view on the measured dental cavity; (**c**) OCT B-scan, where the depth and the width of the cavity are measured; (**d**) volumetric OCT reconstruction, on which the width and the length are measured.

**Figure 16:**
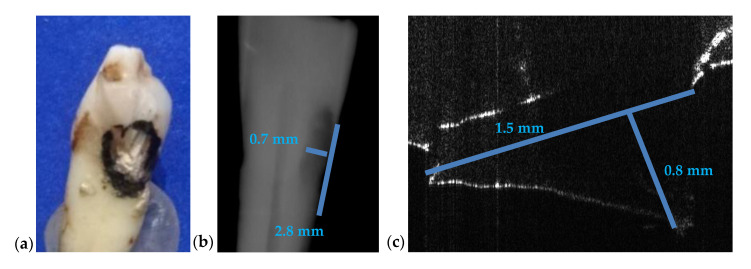
(**a**) Incisive tooth with an area marked for OCT investigations; (**b**) a section cropped from the intraoral radiography with measurements of the cavity; (**c**) OCT B-scan where the depth and the length measurements of the cavity have been performed.

**Figure 17:**
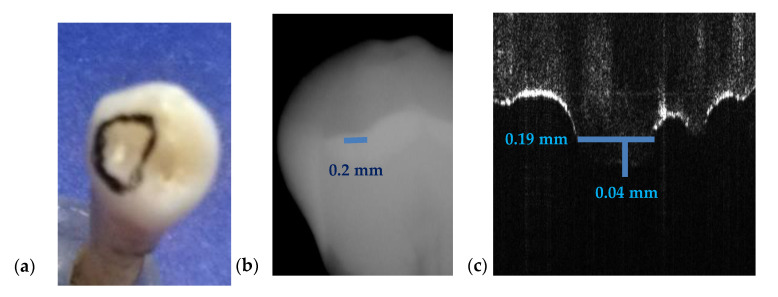
(**a**) Canine tooth, with an area marked for OCT investigations; (**b**) a section cropped from the intraoral radiography where the cavity was measured; (**c**) OCT B-scans with measurements of depth and width of the cavity.

A few relevant examples of cavities are considered to make such a comparison in Figure 15, Figure 16 and Figure 17, which show that measurements on intraoral radiographs give results different than those on OCT images.

For the case presented in Figure 15, there is a difference of 0.9 mm in length and the depth is double on the intraoral radiograph as compared to the OCT image.

For the case presented in Figure 16, the difference between the measurements of the length on both images is 1.3 mm while the difference for depth measurements is 0.1 mm. Measurements errors from Figure 15b and Figure 16b are therefore significant. We point out that the values obtained using OCT images are the correct ones because they have been checked by direct measurements.

The case presented in Figure 17 consists of a small cavity that is barely visible on intraoral radiography. The fact that an estimation using the radiograph gives only 0.01 mm in difference from the value obtained using the OCT image is merely a coincidence. There is no way to correctly measure the cavity on an intraoral radiograph (in depth, length, or width) while Figure 17c proves that on the OCT B-scan one can properly perform measurements.

We may conclude that cavities can be identified and measured with both techniques but can be correctly assessed in terms of their dimensions using OCT only. Furthermore, there are new (early) cavities that cannot be observed on radiographs, but that can be spotted on OCT images—as concluded in our preliminary work in [17]. Intraoral radiographs and OCT images can be both utilized for diagnosis of dental cavities but the most accurate method for quantitatively assessing dental cavities proves to be OCT. Histopathology, which is the gold standard in microscopic examinations, could have been an option to compare with both radiography and OCT. However, in this case we address only dimensional measurements on (the surface of) teeth, and not cell-level evaluations of tissue.

Following these ascertainments, a comparison has been made between the measuring accuracy of the different dimensions of the detected cavities, using the two methods. The results are presented, for the examples considered in the paper, in Table 1.

One can observe that there is no column *width* for the *radiography* assessment, because for measuring cavities intraoral and panoramic radiographs are utilized, and they are 2D images. The relative error

(1) $$\varepsilon \;\left(\%\right)=\frac{\left|x_{Radiography}-x_{OCT}\right|}{x_{OCT}}\cdot 100$$

is calculated in all the cases where data has been available with both imaging techniques, where $x_{Radiography}$ is the length or depth measured on radiographs and $x_{OCT}$ is the length or depth measured on OCT images.

**Table 1:** Measurements performed on both radiography and OCT images obtained for the same tooth.

Measurements on Tooth From	Radiography		OCT			Relative Error ε (%)	
	Length (mm)	Depth (mm)	Length (mm)	Width (mm)	Depth (mm)	For Length	For Depth
Figure 7	3.2	1.6	1.9	0.8	0.5	68	110
Figure 8	2.4	2.2	3.0	2.7	3.3	20	33
Figure 9	1.5	1.9	2.0	0.9	3.3	75	42
Figure 10	2.7	3.0	3.5	3.9	3.8	34	21
Figure 15	2.9	0.8	2	1.1	0.4	45	100
Figure 16	2.8	0.7	1.5	-	0.8	86	12
Figure 17	0.2	-	0.19	-	0.04	5	-
**Mean Relative Error** $\bar{\varepsilon }\;$ **(%)**		$\bar{\varepsilon }=\frac{\sum_{1}^{N}\varepsilon_{j}}{N}=50\%$,					(2)
		where *N* = 13 is the number of relative errors for measurements performed with both methods.					
**Standard Deviation of Relative Errors σ (%)**		$\sigma =\sqrt{\frac{\sum_{1}^{N}{\left(\varepsilon_{j}-\bar{\varepsilon }\right)}^{2}}{N-1}}=34.3\%$					(3)

For these errors, the mean value and the standard deviation are calculated in Table 1. One can see the quite large value of the mean, due to the large errors made in radiographic measurements (on images that do lack resolution, but also contrast). There is also a large standard deviation for these relative errors, because some measurements can be made more precisely on radiographs, while others are significantly flawed. We may conclude that only OCT images present enough resolution to allow for such accurate assessments.

Two imaging characteristics that must be discussed are contrast and sharpness. To compare images obtained with two different methods, the measurements are performed using a single software. The differences between the images of each sample can be quantified by analyzing the data provided by the Romexis software. Table 2 presents the values gathered for each of the above 2D image. One must remark that 3D CBCT images are adjustable in terms of contrast and brightness from 0 to 4095, and sharpness from 0 to 10.

*The contrast* is calculated with [1]

(4) $$C=(I_{max}-I_{min})/(I_{max}+I_{min}),\;$$

where $I_{max}$ and $I_{min}$ are the maximum and minimum pixel intensity, respectively.

*The Contrast-to-Noise Ratio* (CNR) can be also calculated using the equation [1]

(5) $$CNR=\frac{\left|I_{min}-I_{max}\right|}{\sigma_{0}}$$

where $\sigma_{0}$ is the standard deviation of the pixel intensity *I*, and it is provided by the imaging software. Their values are provided in Table 2 for each of the two methods, comparatively, when both are available for a certain sample investigated in this study (and only for one of the methods, when only one of them is available). This comparison allows for calculating the relative error for each sample and parameter, like the calculus performed in Table 1 for the measured dimensions of the cavities. A mean relative error and its standard deviation can be then obtained for each of the two parameters (Table 2).

**Table 2:** Measurements of Contrast (C) and Contrast-to-Noise Ratio (CNR) performed on radiography and OCT images obtained for each sample considered in the study.

Sample from Figure	Imaging Method	Maximum and Minimum Pixel Intensity *I*		$\sigma_{0}$ (%)	C	$\varepsilon_{C}^{j}$ (%)	CNR	$\varepsilon_{CNR}^{j}$ (%)
		$I_{max}$	$I_{min}$					
7	OCT	255	1	71.7	0.992	98.4	3.54	4.1
	Panoramic	192	64	37.57	0.5		3.4	
8	OCT	255	1	92.25	0.992	41.7	2.73	13.8
	Panoramic	208	31	55.7	0.74		3.17	
9	OCT	255	1	53.82	0.992	86.8	4.71	54.9
	Panoramic	193	59	44	0.531		3.04	
10	OCT	255	2	55.15	0.984	-	4.58	-
11	OCT	255	5	45.42	0.961	45.1	5.5	27.6
	Panoramic	123	25	22.7	0.662		4.31	
12	OCT	255	0	45.25	1	51	5.63	30.6
	Panoramic	123	25	22.7	0.662		4.31	
13	OCT	255	9	94.76	0.931	37.7	2.59	33.7
	Panoramic	238	46	49	0.676		3.91	
14	OCT	255	4	89.8	0.969	-	2.79	-
15	OCT	188	0	26.7	1	16.8	7.04	55.1
	Panoramic	232	18	47.11	0.856		4.54	
16	OCT	255	0	44	1	16.8	5.79	27.5
	Panoramic	232	18	47.11	0.856		4.54	
17	OCT	255	2	70.3	0.984	14.9	3.59	20.9
	Panoramic	232	18	47.11	0.856		4.54	
**Mean Relative Error of *C* (** ${\bar{\varepsilon }}_{C}$ **)**			${\bar{\varepsilon }}_{C}=\frac{\left(\sum_{1}^{N}\varepsilon_{C}^{j}\right)}{N}=45.46\%$,					(6)
			where *N* = 9 is the number of relative errors for measurements performed with both methods.					
**Standard Deviation of the Relative Errors of *C* (** $\sigma_{C}$ **)**			$\sigma_{C}=\sqrt{\frac{\sum_{1}^{N}{\left(\varepsilon_{C}^{j}-{\bar{\varepsilon }}_{C}\right)}^{2}}{N-1}}=29.9\%$					(7)
**Mean relative Error of *CNR* (** ${\bar{\varepsilon }}_{CNR}$ **)**			${\bar{\varepsilon }}_{CNR}=\frac{\left(\sum_{1}^{N}\varepsilon_{CNR}^{j}\right)}{N}=29.8\%$,					(8)
			where *N* = 9 is the number of relative errors for measurements performed with both methods.					
**Standard Deviation of the Relative Errors of *CNR* (** $\sigma_{CNR}$ **)**			$\sigma_{CNR}=\sqrt{\frac{\sum_{1}^{N}{\left(\varepsilon_{CNR}^{j}-{\bar{\varepsilon }}_{CNR}\right)}^{2}}{N-1}}=16.93\%$					(9)

From Table 2, the difference in contrast between OCT images and radiographs is significant, with a mean relative error of 45.46%. This means that OCT images have better contrast. This was expected because OCT images are performed directly on the tooth, while for radiographs there are other anatomical elements (i.e., bone, gingiva, tongue, cheek, jaw, lips, etc.) that appear on the image and influence the contrast. The mean difference between the contrast values of OCT images and radiographs from cases where radiographs were performed on patients (Figure 7, Figure 8 and Figure 9) is 0.40, while the mean difference in the cases where radiographs were performed on extracted teeth (Figure 15, Figure 16 and Figure 17) is 0.13. This proves that soft and hard tissues existing around the tooth influence the contrast.

The mean relative error of CNR is 29.8%, and it is smaller than the value of the mean relative error of contrast. This means that images have good sharpness, even if in several OCT images (Figure 10, Figure 13 and Figure 17) and radiographs (Figure 13, Figure 15 and Figure 16), different artefacts are visible. Any metallic surface or a material that has great reflexivity is producing artefacts when OCT investigations are performed around that area. Additionally, metallic surfaces influence contrast and sharpness of radiographs because they absorb X-ray radiation; thus, sometimes image reconstruction artefacts appear. Figure 13 is an example of such a situation where the metallic cape produces artefacts on both OCT images and radiographs.

### 3.4. Treatment Assessments Using OCT

**Figure 18:**
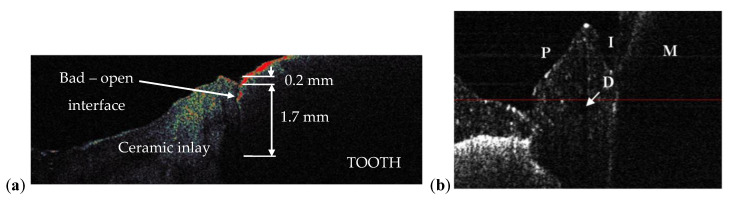
(**a**) OCT B-scan of a treated dental cavity obtained with an in-house developed 1D GS-based OCT handheld probe [30,48], which allows for the evaluation of the interface between a tooth and the ceramic inlay: apparently good interface, closed, but with a crack between tooth and inlay; (**b**) OCT B-scan of a metal ceramic dental prosthesis using the same 1D GS-based handheld probe and an SD-OCT system [48], with the following notations: M, 1st molar (M); P, 1st premolar; D, defect in the ceramic layer; I, interface between M and P.

**Figure 19:**
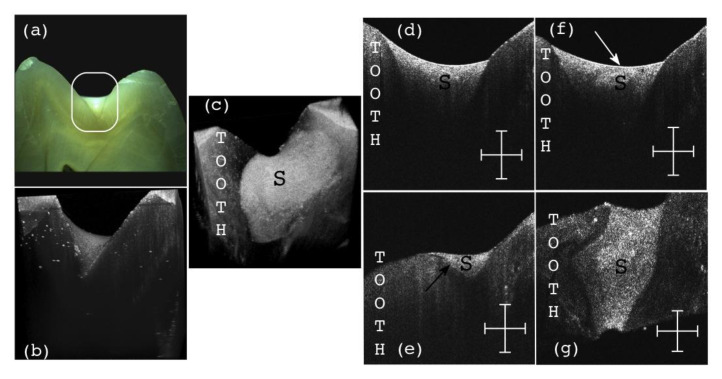
OCT B-scan of a treated dental cavity: (**a**) area of interest; (**b**) 3D OCT reconstruction after the investigation showing the mentioned area; (**c**) general aspect of the sealant (S) from the occlusal view; (**d**) B–scan of the structure presenting a good interface between the tooth enamel and the sealant (S); (**e**) B–scan presenting an open interface (black arrow) between the sealant (S) and the tooth structure; (**f**) B–scan presenting a defect (white arrow) inside of the sealant material; (**g**) C–scan of the structure presenting no defects inside the sealant (S) material at the considered depth;. Scale bars: 1 mm.

**Figure 20:**
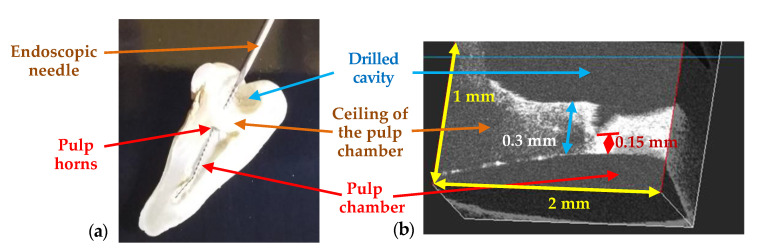
(**a**) Tooth morphology, sectioned after the procedure, showing the Remaining Dental Thickness (RDT) between the drilled cavity and the pulp chamber. An endodontic needle is inserted through the drilled cavity towards the pulp chamber, via the pulp horns, therefore the drilling process has affected the pulp chamber. (**b**) Real-time OCT evaluation of the RDT, showing its decrease to a critical value, for which a fracture occurs-from the detailed study in [55].

Three examples on the capability of OCT to perform dental treatment assessments are provided in Figure 18, Figure 19 and Figure 20. Numerous other such examples have been reported in our previous studies [47]. Such applications have also been considered from the late 1990s, including in early studies of OCT in dentistry [10,11].

The most common dental treatments that can be targeted using OCT are related to cavities. As shown in Figure 18a, an OCT B-scan (i.e., a cross-section inside the teeth) can reveal defects both in the inlay introduced in the dental cavity and in the interface between the tooth and the added inlay. The capability of OCT in this respect is unique: an interface defect may not appear on the tooth surface or it may look superficial, as in Figure 18a. However, using the non-invasive IR laser-based OCT investigation, one remarks on the OCT B-scan that the (open) interface has not just a 0.2 mm surface defect, but a (precisely evaluated) 1.7 mm depth defect. The latter would go unnoticed if it were not for the OCT investigation, thus becoming a source of secondary cavities.

The question is: can such an assessment be made using the common (and most utilized) radiography? The answer is negative, as we have demonstrated in detail in [31]. On the other hand, OCT can perform this task, as both the necessary resolution and penetration depths are fully within its capabilities. An example in this respect is shown in Figure 19, in an investigation similar to those in [31]: ex vivo, on half of a tooth, sectioned and observed with optical microscopy in Figure 19a. Early cavities cannot be measured (and some cannot even be remarked) on radiographies, nor can be assessed issues of the dental treatment. In contrast, OCT allows for such an evaluation, as shown in Figure 19 on the entire 3D OCT reconstruction Figure 19b, on its oclusal view Figure 19c or using B-scans, as in the Figure 19d–f sections. Another view of interest available with OCT is in the *en-face* image/C-scan, such as the one in Figure 19g, obtained from the 3D OCT image by sectioning it with a plane situated at a certain (constant) depth—in this case from the occlusal surface of the tooth. Using the MS enhanced OCT technique [7], such *en-face* OCT images can be obtained directly, without having to retrieve 3D/volumetric reconstructions first. They can be used to obtain a more complete view on the location and extension of defects, as detailed in [31] as well.

Other aspects can be revealed as well, using OCT, for example regarding the nature of the material of the sealant (S) utilized for the cavity treatment. A ceramic inlay can thus be clearly seen differently from a polymeric one because of their different porosity [49].

Furthermore, OCT investigations can be also performed in vivo, using handheld scanning probes. Such probes have been developed using 2D GSs [50,51], 2D Micro-Electro-Mechanical Systems (MEMS) [52,53], or, for Dental Medicine, even a simple 1D GS (lower-cost and light-weight). Such a 1D GS-based handheld probe [30,49]) can be utilized for such applications even if it provides only a single B-scan at the time, because the dentist is providing the second direction of lateral scanning by moving the probe across the area of interest (for example the tooth surface), thus monitoring in real time on a PC screen successive B-scans. The dentist can thus assess a performed treatment by sweeping the tissue and observing (in vivo, non-invasively, and in real time) the various cross-sections beneath the observed tissue surface. Once a defect inside the S or at the tooth/S interface is identified—as in Figure 18a and Figure 19e,f—it must be corrected. Such defects left untreated/uncorrected (or even undetected, if only radiography is utilized) become sources of secondary cavities, filled (as it is well-known) with anaerobic bacteria, thus becoming a more severe dental issue than open-surface dental cavities.

Beside tooth treatments, dental crowns can and should also be investigated/verified prior to being placed in the mouth, to detect eventual inner defects (D), as those shown in Figure 18b. Such defects are sources of cracks, that usually occur (even) within weeks after placing the crown in the patient’s mouth, situation that should be avoided.

Among the sources of defects such as those in Figure 18b one has the loss of calibration of the dental ovens where metal ceramic or all ceramic crowns are sintered. We have studied, for the first time to our knowledge, using OCT [54,55], this loss of calibration that produces a lower or higher than normal sintering temperature of the ceramics. OCT has thus been demonstrated to provide both qualitative [54], but, more importantly, quantitative results [55] in assessing the maximum temperature reached in the oven and its difference from the temperature prescribed by the manufacturer (for each specific material). Rules-of-thumb have been thus extracted in [54] from both OCT C-scans and reflectivity profiles extracted from them, as well as from related parameters obtained from these profiles [55].

Finally, besides assessing (using OCT) performed treatments, the imaging technique can be utilized during certain dental procedures as well. An example is presented in Figure 20, from the detailed study in [56]: OCT B-scans are retrieved during the drilling process of a dental cavity (ex vivo). Using only visual observation the dental practitioner may find difficult to avoid breaking the ceiling of the pulp chamber, while using OCT the remaining dental thickness (RDT) of this ceiling can be kept above the safety limit of approximately 0.5 mm [56]. In Figure 20 the (unwanted) situation of opening the pulp chamber is presented. In the B-scan in Figure 20b the moment when the fracture of the remaining dentin occurs is captured, in real time. Supervising the drilling process with OCT may prevent the need for an endodontic undesired treatment, by maintaining the tooth integrity.

### 3.5. Sinergy between Radiography and OCT

There are four aspects that differentiate radiography and OCT in terms of imaging quality: image resolution, penetration depth, field-of-view (FOV), and radiation safety. In terms of penetration depth and FOV, radiography is clearly superior to OCT. In terms of resolution, OCT is superior, and it is also radiation-free (while the level of radiation not only in a radiography imaging session, but also during all phases of a dental treatment is an issue of concern for patients, as well as professionals [8,9]). However, potentially, the performance of OCT may be affected by artefacts, especially due to involuntary movements of the patient. The choice of the OCT system and of its performances must be also carefully made, to provide the necessary acquisition speed and video-frame imaging capabilities for real time, in vivo imaging, the latter using, for Dental Medicine, dedicated handheld probes [50,51,52,53], as demonstrated in [30,49].

According to the level of details and information gathered from an image, the present study has compared OCT and radiography regarding diagnosis or treatments assessment of selected dental issues. For example, a small cavity with a width of 0.2 mm can be observed on a panoramic or on an intraoral radiography but can be correctly assessed (dimensionally) only on OCT images. The results have shown the differences in assessing a cavity and why OCT is the method of choice in the case of small cavities.

OCT also proves useful when details and exact measurements are needed. The drawback of OCT in this case is its limited area of investigation (given its FOV), of less than 5 × 5 mm^2^ (or much smaller, of 1 × 1 mm^2^, for example, when higher resolutions are required). In contrast, panoramic radiography covers the whole mouth. This means that it takes time to investigate all teeth with OCT, while a panoramic radiograph only takes 15 s of exposure.

Crown and filling adaptation are other dental aspects that are covered by both techniques. For dental fillings, OCT and 3D CBCT can be at the same level because the adaptation can be correctly assessed on both 3D renderings, with a better resolution of OCT (i.e., commonly 4 to 10 µm axial resolution for OCT, and 75 to 150 µm for radiography). The drawback of radiography is the fact that when the dental crown is made of a radiopaque material such as metal, artefacts appear on images. OCT can provide accurate images of the surface of metallic objects, but it cannot penetrate the material. Therefore, when assessing the adaptation of metallic dental crowns, OCT images can offer qualitative information and details, while radiographs (both panoramic and 3D CBCT) have reconstruction artefacts because of the major amount of metal from the dental crown.

To summarize the results, in Table 3 one can see what type of medical imaging technique is more suitable to be utilized to diagnose or to assess the proper treatment for selected dental issues.

A synergy between radiography and OCT can be concluded from the study—as shown in Table 4. First, the two methods can validate each other to some extent, as some dental issues can be investigated with both imaging techniques, including cavities, periodontitis, and adaptation of dental crowns or dental fillings. Dental issues can be spotted and assessed on images gathered from both techniques, but with differences at the level of details and regarding the amount of information that can be observed on both images—as observed in the examples considered in this study.

Secondly, OCT and radiography are complementary, as there are dental issues that cannot be investigated with OCT and others that cannot be investigated with radiography. Essentially, for large areas and for investigating in depth the sample, it is better to choose X-ray techniques, while for accurate high-resolution images of small area of the surface and up to 2 mm in depth of the sample, it is better to choose the OCT technique.

## 4. Conclusions

The study has considered various applications of dental medicine, comparing the performance of radiography and OCT in assessing dental affections and in treatment monitoring. The reciprocal validation and the complementarity of the two imaging techniques have been studied. The contrast and possible synergy of radiography and OCT can be a beautiful example of Niels Bohr’s principle: “Contraria non contradictoria, sed complementa sunt”.

Dental issues assessed with radiography are bone analysis, surgery monitoring (i.e., bone augmentation and implant insertion), apical infections, or root canal filling. OCT can be utilized when there are gingiva issues, enamel or dentine problems (i.e., deformations, demineralization, or cracks), early stage cavities, or metallic dental crowns; also, for precise measurements of dental issues (i.e., cavities, including early ones) and for monitoring dental drilling during the procedure. One must also point to other techniques that may serve to cover specific (niche) applications, including Scanning Electron Microscopy (SEM) for small details in (cleaning) the apical canal, for example, micro-CT (for superior resolutions than OCT-also providing 3D images) [57], as well as confocal microscopy for research in dental materials, for example. However, radiography remains the common technique for dentistry, while, as pointed out in the study, and considering also cost and availability, OCT can become a daily-basis imaging technique in dentistry alongside radiography as well. This is also because, although in this study mostly ex vivo OCT investigations have been considered (to assess resolution and penetration capabilities), OCT imaging can be also performed in vivo, as demonstrated using 1D GS- [30,49], 2D GSs- [50,51], and 2D MEMS-based [52,53] handheld scanning probes.

Advantages and drawbacks of using one technique or the other should however be considered carefully for each specific application. This study may contribute to serve as a guidance in this respect. Considering all aspects (i.e., image resolution/level of detail, time consuming criterion, accuracy, artefacts, area of investigation/FOV, and radiation issues/invasiveness), one can thus select the most suitable medical imaging technique for a certain dental issue or determine that both are needed for a full clinical assessment.

## Figures and Tables

**Table 3:** Medical imaging technique suitable for selected dental issues.

Dental Issue	Radiography	OCT
Cavities	Cavities smaller than 0.5 × 0.5 mm are barely visible on any type of radiography	Correct quantitative assessment of small cavities (Figure 7, Figure 8, Figure 9, Figure 10, Figure 15, Figure 16, Figure 17)
Dental crowns (metal ceramic or all ceramic)	Artefacts may appear therefore the obtained images cannot be utilized	Accurate surface images for metallic parts; high-resolution images beneath the sample surface for non-metallic (ceramic or polymer) crowns
Orthodontics	Appropriate to measure/observe teeth movement	Accurate for tooth analysis (i.e., for enamel and dentine–Figure 11, Figure 12 and Figure 14)
Bone issues assessment	Accurate investigations of bone density and quantity assessment on 3D CBCT images (see the example in Figure 3)	Cannot penetrate through the bone more than 1 to 2 mm
Periodontitis	The disease can be monitored during the treatment (example, Figure 5)	Exact measurements of bone loss/gain are possible
Crown/filling adaptation	High-quality images for materials that do not absorb X-ray radiation in excess	High quality images for most types of materials used in dentistry (Figure 13)
Enamel/dentine issues	Not visible on any type of radiography	Qualitative images, but also quantitative evaluations can be obtained-even beneath the teeth surface
Soft tissue	Not visible using any type of radiography	Qualitative images can be obtained. Depth limitation of up to 2 mm.

**Table 4:** Medical imaging technique suitable for diagnose/treatment checking and assessment of dental issues.

Dental Issue	Diagnose/Treatment Monitoring	Measuring Capability of Their Spatial Extension
Cavities	X-ray and OCT	OCT
Metal crowns	OCT	OCT
Orthodontics	X-ray and OCT	OCT
Bone assessment	X-ray	X-ray
Periodontitis	X-ray and OCT	X-ray and OCT
Crown/filling adaptation	X-ray and OCT	OCT
Enamel/dentine issues	OCT	OCT
Soft tissue	OCT	OCT
